# The impact of disease-related impairments on disability and health-related quality of life: a systematic review

**DOI:** 10.1186/1471-2288-7-24

**Published:** 2007-06-19

**Authors:** Nadine Weisscher, Rob J de Haan, Marinus Vermeulen

**Affiliations:** 1 Department of Neurology, Academic Medical Center, University of Amsterdam, Amsterdam, The Netherlands; 2 Department of Clinical Epidemiology and Biostatistics, Academic Medical Center, University of Amsterdam, Amsterdam, The Netherlands

## Abstract

**Background:**

To investigate the interchangeability of measures of disability and health-related quality of life (HRQL) by comparing their associations patterns with disease-related impairment measures in patients with a variety of conditions.

**Methods:**

A systematic literature search of MEDLINE, EMBASE, Web of Science and a hand search of reference lists through January 2006. Studies were included if they reported associations patterns between impairment and disability and between impairment and HRQL. Correlation coefficients were transformed to Fisher's *z *effect size (ES(*z*)). Weighted averages were reported as pooled ES(*z*) with 95% confidence intervals (CI).

**Results:**

The relationship between impairment and disability was stronger (pooled ES(*z*) = 0.69; 95% CI, 0.66 – 0.72) than between impairment and HRQL (pooled ES(*z*) = 0.38; 95% CI, 0.36 – 0.41). The physical component score (pooled ES(*z*) = 0.43; 95% CI, 0.39 – 0.47) and disease-specific HRQL (pooled ES(*z*) = 0.46; 95% CI, 0.40 – 0.51) were stronger associated with impairments than the mental component score (pooled ES(*z*) = 0.28; 95% CI, 0.20 – 0.36) and generic HRQL (pooled ES(*z*) = 0.36; 95% CI, 0.33 – 0.39).

**Conclusion:**

This study shows measures of disability and different HRQL domains were not equally related to impairment. Patient's impairments are better reflected in disability measures, than in HRQL instruments. There are many outcomes of interest and precisely defining them and measuring them will improve assessing the impact of new interventions.

## Background

Choosing an outcome measure for use in clinical research is a complex process. If the outcomes are chosen inappropriately, a study may provide unreliable results [1]. Frequently used outcomes measures are mortality rates, number of events (recurrent myocard infarction) or disease activity (lesion load on MRI).

Besides biological measures other levels of clinical measurement can be considered in clinical studies: impairments, disability and health-related quality of life (HRQL). Impairments are the direct organic manifestations of the disease such as consciousness and paresis [2,3]. Disability can be defined as limitations in carrying out activities of daily living, such as self-care, mobility and activities inside or outside the home [2,3]. HRQL refers to a broad spectrum of consequences of disease. Although this concept also includes elements of impairments and disabilities, it has a strong focus on patient's social functioning and perceived health status and well being [4,5]. Hence, impairment measures are closely related to the patient's disorder, whereas the other outcome measures focus on the patient's level of functional health. Each subsequent level of clinical measurement is increasingly less disease-specific and more relevant to the patient.

Recently, patient-relevant outcomes in terms of disability and HRQL have become more important [6]. Although both functional outcomes reflect the consequences of diseases on personal level, they are conceptually different and not synonymous as is often thought by clinical researchers. Consequently, the decision to use one of these measures can have important implications for the interpretation of the study results. The objective of this systematic review is to investigate the interchangeability of measures of disability and HRQL by comparing their association patterns with disease-related impairment measures.

## Methods

### Data sources

We conducted a systematic literature search of MEDLINE from 1966 through January 2006, EMBASE from 1980 through January 2006 and Web of Science from 1988 through January 2006 to identify studies addressing the relation between outcome measures. A search strategy using Medical Subject Heading, text words and Publication Types *Impairment *or *Body function *(Publication date from 2001) or *Body structure *(Publication date from 2001) and *Disability (Evaluation) *or *Activity *or *Disabled Persons *or *Activities of Daily Living *and *Health-related quality of life *combined with *Association *or *Evaluation Studies *or *Comparative Study *or *Validation Studies *was used. Studies from English, German and French literature were included. The electronic search was supplemented by hand searching the investigators files, and retrieval of references cited in available literature. The search strategy was composed by one of the authors (NW) in consultation with a clinical librarian.

### Study selection

A set of explicit criteria, composed by three investigators (NW, RdH, MV), were used for selection of the literature. Articles were included if they focused on methodological or metric aspects of patient-based outcomes (for example, methods of evaluating such measures, psychometric or clinimetric assessment of measures, comparative studies of measures).

Studies were included in which both the association between impairment and disability, and between impairment and HRQL were calculated by means of correlation coefficients or other association measures, regardless of the disease with the exception of psychiatric disorders. Hence, to improve the comparability between the correlation patterns between the different health concepts, only studies were included in which the three types of health outcomes were assessed in the same patient population. The measurement instruments were questionnaires or observation lists. When a multi-scale questionnaire focuses on more than one concept we only included the sub-scale for the health domain in question. In case of double publication we selected the first publication in time. Excluded were studies which valuated HRQL in terms of utilities [7] or composite scores of conceptual different measures.

### Data extraction

All data were independently abstracted by two investigators (NW, RdH) through use of a list of the criteria composed by three investigators. After study selection, measures were categorized according the definitions of the WHO *International Classification of Impairments, Disabilities, and Handicap *version [2] and the revised *International Classification of Functioning *[3]. Outcome measures were classified into one of the following categories: impairment, disability, and HRQL. The latter was further categorized into generic HRQL, disease-specific HRQL, mental component of HRQL and physical component of HRQL. When there was doubt on the classification of the health domain a third reviewer (MV) was consulted.

A first draft synthesizing the data was produced by the first author of this review (NW) and extensively criticized by two other authors. The following information was abstracted: author, year of publication, sample size, disease, type of impairment and disability scales, distinction between generic or disease-specific and mental or physical HRQL, and the associations with impairment. Studies reporting repeated measures only baseline data was abstracted. Disagreements in abstracting were solved by discussion.

### Statistical analysis

The correlations coefficients (r) published were converted using Fischer's variance stabilizing *z *transform (ES(*z*)) [8].

ES(*z*) = 1/2 log_e _[1+r/1-r]

were the asymptotic variance was:

*v *_ES(*z*) _= 1/n-3

Mean ES(*z*) were calculated for studies reporting multiple correlations between the concepts studied [9]. The ES(*z*) were combined using a fixed or random effects model, when appropriate. The weight for each study was inversely proportional to the square root of the number of patients included in the study. The methods for obtaining the pooled ES(*z*) and its 95% confidence interval (CI) are described in detail elsewhere [10]. We labeled the strength of the ES 0.20 as small, 0.50 as medium and 0.80 as large [11]. The pooled ES(*z*) were converted back to a correlation coefficient [12]:

*r *= tanh(ES(*z*))

in which we labeled the strength of the association; absolute values of r, 0.00–0.19 is regarded as very weak, 0.20–0.39 as weak, 0.40–0.59 as moderate, 0.60–0.79 as strong and 0.80–1.00 as very strong correlation [13]. The backward transformed correlation coefficients were also expressed in variance explained (r^2^). The square of the correlation coefficient tells us what proportion of the variance of one variable can be explained by the linear correlation with the other.

Statistical heterogeneity was measured using the Cochrane Q statistics; *p *≤ 0.10 was considered representative of significant statistical heterogeneity. To establish the effect of heterogeneity between studies on meta-analysis conclusions, we performed several subgroup analyses: impairment in relation to generic or disease-specific HRQL and impairment versus the physical or mental aspects of HRQL.

We constructed normal quantile plots to assess whether the ES(*z*) estimates are (approximately) normal distributed and to search for publication bias [14]. With regard to the presence of publication bias we also used the Spearman's rho rank correlation method [12] to test the relationship between the standardized ES(*z*) and sample size (*p *< 0.05 indicates significant publication bias) and a fail-safe number approach. A fail-safe number is the number of non-significant, unpublished, or missing studies that would need to be added to the meta-analysis in order to change the results of the analysis. If this number is relative large to the number of observed studies, the observed results, even with some publication bias, can be treated as a reliable estimate of the true effect [15]. All analyses were performed in Meta-Win, version 2.0.

## Results

### Study characteristics

**Figure 1:**
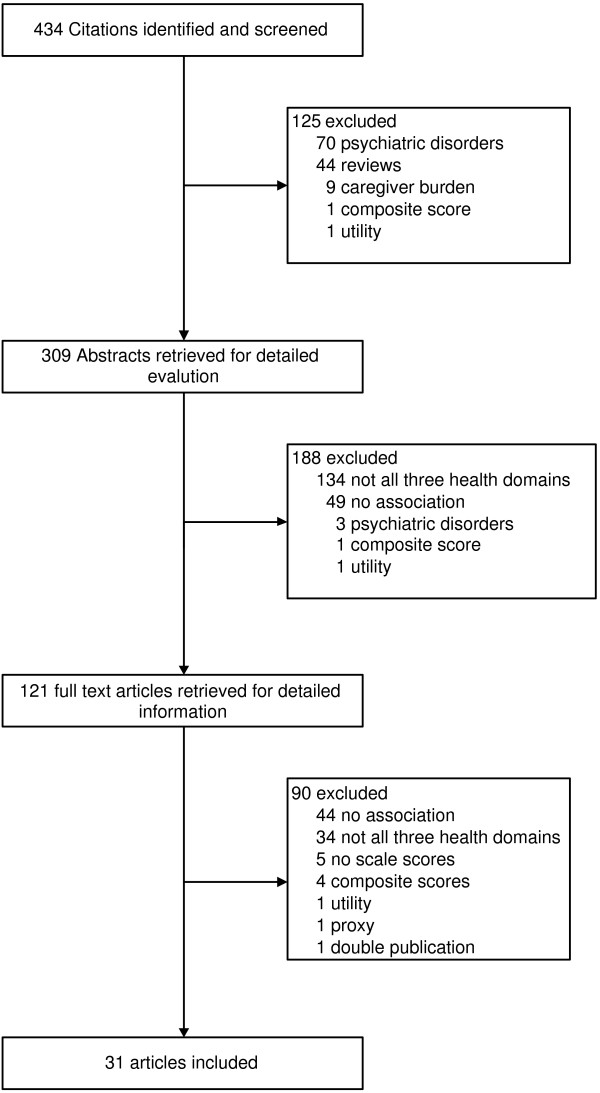
Summary description of the studies included and excluded in the meta-analysis.

Our initial search yielded 434 potentially literature citations (Figure 1). Of these, 125 were excluded based on title (70 were investigating a psychiatric disorder, 44 were review articles, nine articles measured caregiver burden, one article reported data about a composite score and one reported HRQL in terms of utilities). Abstracts from 309 articles were retrieved and additional 188 articles were excluded (134 studies did not measure all three health domains, 49 did not report concrete associations between the health domains, three were investigating a psychiatric disorder, one reported data about a composite score and one reported HRQL in terms of utilities), leaving 121 articles for full review. Of these, 90 were excluded (44 did not report association patterns, 34 articles measured not all three domains, in five articles impairment measures were no scale score, four articles used composite scores, one reported utility based HRQL, one concerned a proxy measure and one a double publication).

A total of 31 studies, with an average sample size of 401 (range 38 – 6497) persons, were found to conform to our inclusion criteria [16-46] (Table 1). Seven studies reported data on outcome measures in patients with arthritis, six on Multiple Sclerosis, three on Parkinson's disease, two on stroke, two on spinal cord injury, four on a variety of joint diseases (knee, patella, elbow, shoulder), one cervical dystonia, one diabetic neuropathy, one facial nerve paralysis, one AIDS, one chronic dysphonia, one chronic obstructive pulmonary disease, and one on psoriasis.

**Table 1:** Information on the 31 studies included in the analysis ordered on disease

**References**	**N**	**Disease**	**Impairment**	**Disability**	**HRQL**	
					**Generic**	**Specific**

Deyo, 1983 [22]	97	Arthritis	Morning stiffness Grip strength	SIP physical ARA	SIP total SIP psychosocial	
Birrell, 1998 [19]	86	Arthritis	OSRA	HAQ	SF-36	
Kvien, 1998 [29]	1030	Arthritis	VAS pain VAS fatigue	MHAQ	SF-36	AIMS2
Wolfe, 2000 [45]	1773	Arthritis	Global GI severity	HAQ	SF-36	
Angst, 2004 [16]	43	Arthritis	cASES	pASES DASH	SF-36	
Angst, 2005 [17]	79	Arthritis	cASES	pASES DASH	SF-36	
Salaffi, 2005 [39]	244	Arthritis	WOMAC-Pain WOMAC-Stiffness	WOMAC-Function	SF-36	
Rothwell, 1997 [37]	42	Multiple sclerosis	EDSS	OPCS	SF-36	
Sharrack, 1999 [42]	50	Multiple sclerosis	EDSS SNRS	Barthel Index FIM CAMBS dis. AI	SF-36 EuroQol VAS	
Schwartz, 1999 [41]	274	Multiple sclerosis	SI	Disease steps AI	HSQ	
Miller, 2000 [34]	300	Multiple sclerosis	EDSS MSFC	SIP physical	SIP psychosocial SF-36	
Hobart, 2000 [26]	64	Multiple sclerosis	EDSS	Barthel Index FIM	SF-36 Well-being GHQ	
Rasova, 2005 [36]	112	Multiple sclerosis	Muscle performance Pulmonary ventilation	Barthel Index		MSQL
Rubenstein, 1998 [38]	193	Parkinson's disease	HY UPDRS motor	FSQ (BADL, IADL)	SF-36	
Schrag, 2000 [40]	79	Parkinson's Disease	H&Y UPDRS motor	PDQ-39 ADL	EuroQol VAS SF-36	PDQ-39
Franchignoni, 2005 [23]	70	Parkinson's Disease	BBS PCS	UPDRS-ADL		PDQ-39
de Haan, 1993 [21]	81	Stroke	Mathew Orgogozo SSS NIHSS CNS	Barthel Index	SIP	
Gottlieb, 2001 [25]	100	Stroke	Clas. Reding	FIM	LSI-a	
Fuhrer, 1992 [24]	140	Spinal Cord Injury	ASIA	FIM	LSI-a	
May, 2002 [33]	98	Spinal Cord Injury	ASIA	FIM		QLI
Marx, 2001 [32]	42	Knee disorders	AAOS Lysholm scale	KOS-ADL	SF-36	
Paxton, 2003 [35]	110	Patellar dislocation	Fulkerson Kujala IKDC Lysholm	Tegner		MFA
MacDermid, 2001 [31]	70	Elbow pathology	ASES pain PREE pain	DASH	SF-36	
Beaton, 1996 [18]	90	Shoulder disorder	Elevation of the shoulder	SST pASES	SF-36 (acute version)	
Lindeboom, 1998 [30]	64	Cervical dystonia	Tsui TWSTRS-P	TWSTRS-D	SF-20	
Vinik, 2005 [44]	262	Diabetic neuropathy	TNS	QOL-DN ADL		QOL-DN
Kahn, 2001 [28]	86	Facial nerve paralysis	FaCE	FDI – physical functioning		FDI – social-well being
Cleary, 1993 [20]	189	AIDS	Extreme pain Total symptom score	FSQ (BADL, IADL)	ORLS Mental Health	
Speyer, 2004 [43]	77	Chronic dysphonia	Voice disorder severity	Impairment daily living		VHI
Hu, 2005 [27]	58	COPD	Breathlessness (VAS)	BDI PSFDQ-Act	SF-36	
Zachariae, 2002 [46]	6497	Psoriasis	PASI Severity	PDI		PLSI

**Abbreviations **(in alphabetic order):

AAOS: American Academy of Orthopaedic Surgeons sports knee-rating scale. AI: Ambulation Index. AIMS2: Arthritis Impact Measurement Scales revised. ARA: American Rheumatism Association's functional classes. ASIA: Total Motor Index Score. (p)cASES: (patient) clinical American Shoulder and Elbow Surgeons questionnaire. BBS: Berg Balance Scale. BDI: Baseline Dyspnea Index. CAMBS: Cambridge Multiple Sclerosis Basic Score. CNS: Canadian Neurological Scale. COPD: Chronic Obstructive Pulmonary Disease. DASH: Disability of the Arm, Shoulder and Hand questionnaire. EDSS: Kurtzke Expanded Disability Scale. FaCE: Facial Clinimetric Evaluation. FDI: Facial Disability Index. FIM: Functional Independence Measure. FSQ: Functional Status Questionnaire (BADL: basic activities of daily life, IADL: instrumental activities of daily life). GHQ: General Health Questionnaire. Global GI severity: global gastrointestinal severity. HAQ: Stanford Health Assessment Questionnaire functional disability index. HSQ: Health Status Questionnaire. H&Y: Hoehn and Yahr. IKDC: International Knee Documentation Committee form. KOS: Knee Outcome Scale. LSIa: Life Satisfaction Index, version A. MFA: Musculoskeletal Function Assessment. MHAQ: Modified Health Assessment Questionnaire. MSFC: Multiple Sclerosis Functional Composite. MSQL: Multiple Sclerosis Quality of life scale. NIHSS: National Institutes of Health Stroke Scale. OPCS: Office of Population Census and Survey disability scale. ORLS: Overall Rating of Life Satisfaction. OSRA: Overall Status measure for Rheumatoid Arthritis. PASI: Psoriasis Area and Severity Index. PCS: Postural Changes Scale. PDI: Psoriasis Disability Index. PDQ-39: Parkinson Disease Questionnaire. PFSDQ-Act: Pulmonary Functional Status and Dyspnea Questionnaire. PLSI: Psoriasis Life Stress Inventory. PREE: Patient-rated Elbow Evaluation. QLI: Ferrans and Powers Quality of life Index. QOL-DN: Norfolk Quality of life Questionnaire-Diabetic Neuropathy. SF-20: Medical Outcome Study 20-Item Short-Form Health Survey. SF-36: Medical Outcome Study 36-Item. SI: Symptom Inventory. SNRS: Scripps Neurological Rating Scale. SSS: Scandinavian Stroke Scale. SST: Simple Shoulder Test. TNS: Total Neuropathy Score. TWSTRS-P(D): Toronto Western Spasmodic Torticollis Rating Scale-Pain(Disability). UPDRS: Unified Parkinson' Disease Rating Scale. VHI: Voice Handicap Index. WOMAC; Western Ontario and McMaster Universities.

The studies included a wide range of disease-related impairment measures, as well as a variety of disability measures (Table 1), in which the Functional Independence Measure (FIM) was the most often used. With regard to the HRQL measures, 17 studies used the SF-36 (or SF-20), 12 studies reported both the physical component and mental component scores, in two studies [37,42] the mental health dimension was considered as mental HRQL. Other scales that were considered as a mental component of HRQL were the psychosocial dimension of the Sickness Impact Profile (SIP) [21,22,34], psychological well-being and the General Health Questionnaire (GHQ) [26], the mental dimension of the Multiple Sclerosis HRQL scale (MSQL) [36], the social well-being dimension of the Facial Disability Index (FDI) [28], the Overall Rating of Life satisfaction (ORLS) [20], and the emotional subscale of the Voice Handicap Index (VHI) [43].

Overall the studies included 86 correlations between impairment and disability and 104 correlations between impairment and HRQL. Five studies reported only one single correlation [24,25,28,33,44], for the remaining studies mean ES(*z*) were calculated for each health domain. Table 2 presents effect sizes of the 31 studies included. Thirteen studies (42%) reported large ES(*z*) between impairment and disability (≥ 0.81), whereas 81% of the ES(*z*) related to HRQL was small to moderate (≤ 0.50). The ES(*z*) between impairment and the different types of HRQL were on average small to moderate, except for disease-specific HRQL; 50% of the studies reported moderate to large ES(*z*) (0.51 – 0.80).

**Table 2:** ES(*z*) of the studies included in the meta-analysis

	**ES(*z*) impairment – disability (n = 31)**	**ES(*z*) impairment – HRQL (n = 31)**			
**References**		***generic *(n = 23)**	***disease-specific *(n = 10)**	***physical *(n = 13)**	***mental *(n = 21)**

Deyo, 1983 [22]	0.23	0.31			0.32
Birrell, 1998 [19]	0.81	0.49		0.65	0.37
Kvien, 1998 [29]	0.50	0.39	0.42		0.41
Wolfe, 2000 [45]	0.35	0.38		0.47	0.29
Angst, 2004 [16]	0.60	0.07		0.29	-0.15
Angst, 2005 [17]	0.60	0.11		0.41	-0.17
Salaffi, 2005 [39]	1.00	0.46		0.41	0.51
Rothwell, 1997 [37]	1.30	0.15			0.05
Sharrack, 1999 [42]	1.05	0.48			0.21
Schwartz, 1999 [41]	0.50	0.68			
Miller, 2000 [34]	1.02	0.18		0.18	0.18
Hobart, 2000 [26]	1.33	0.06		0.34	0.14
Rasova, 2005 [36]	0.56		0.22	0.39	0.04
Rubenstein, 1998 [38]	0.73	0.40		0.48	0.31
Schrag, 2000 [40]	0.66	0.32	0.55	0.49	0.21
Franchignoni, 2005 [23]	0.79		0.71		
de Haan, 1993 [21]	0.87	0.52			0.37
Gottlieb, 2001 [25]	0.55	0.16			
Fuhrer, 1992 [24]	0.85	0.04			
May, 2002 [33]	1.26		0.06		
Marx, 2001 [32]	1.19	0.35		0.83	0.01
Paxton, 2003 [35]	0.35		0.49		
MacDermid, 2001 [31]	0.85	0.41		0.54	0.29
Beaton, 1996 [18]	0.40	0.28			
Lindeboom, 1998 [30]	0.23	0.21			
Vinik, 2005 [44]	0.66		0.59		
Kahn, 2001 [28]	0.87		0.78		0.78
Cleary, 1993 [20]	0.60	0.38			0.38
Speyer, 2004 [43]	1.05		0.55		0.44
Hu, 2005 [27]	0.46	0.45		0.29	0.60
Zachariae, 2002 [46]	0.44		0.40		

### Data synthesis

**Figure 2:**
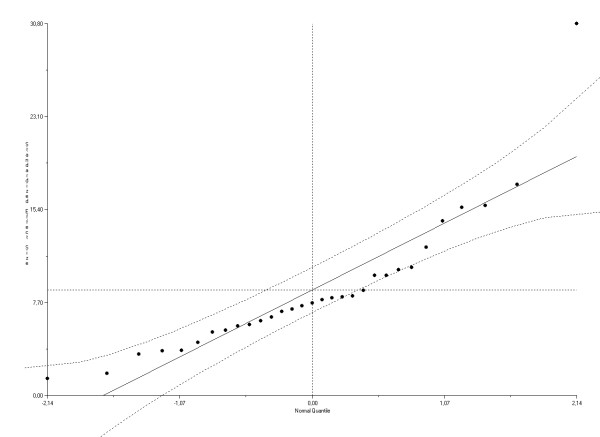
**Normal quantile plot for the ES(*z*) between impairment and disability**. The plot compares two distributions by plotting there quantiles against each other. One of the distributions is the standard normal distribution (X as) while the other is the distribution one wishes to compare it against (here the Y as is defined as ES(*z*)_i_/√_i_). The deviations from linearity indicate deviations in normality. Publication bias will tend to leave strange gaps in the plot or lead to strange curves.

**Figure 3:**
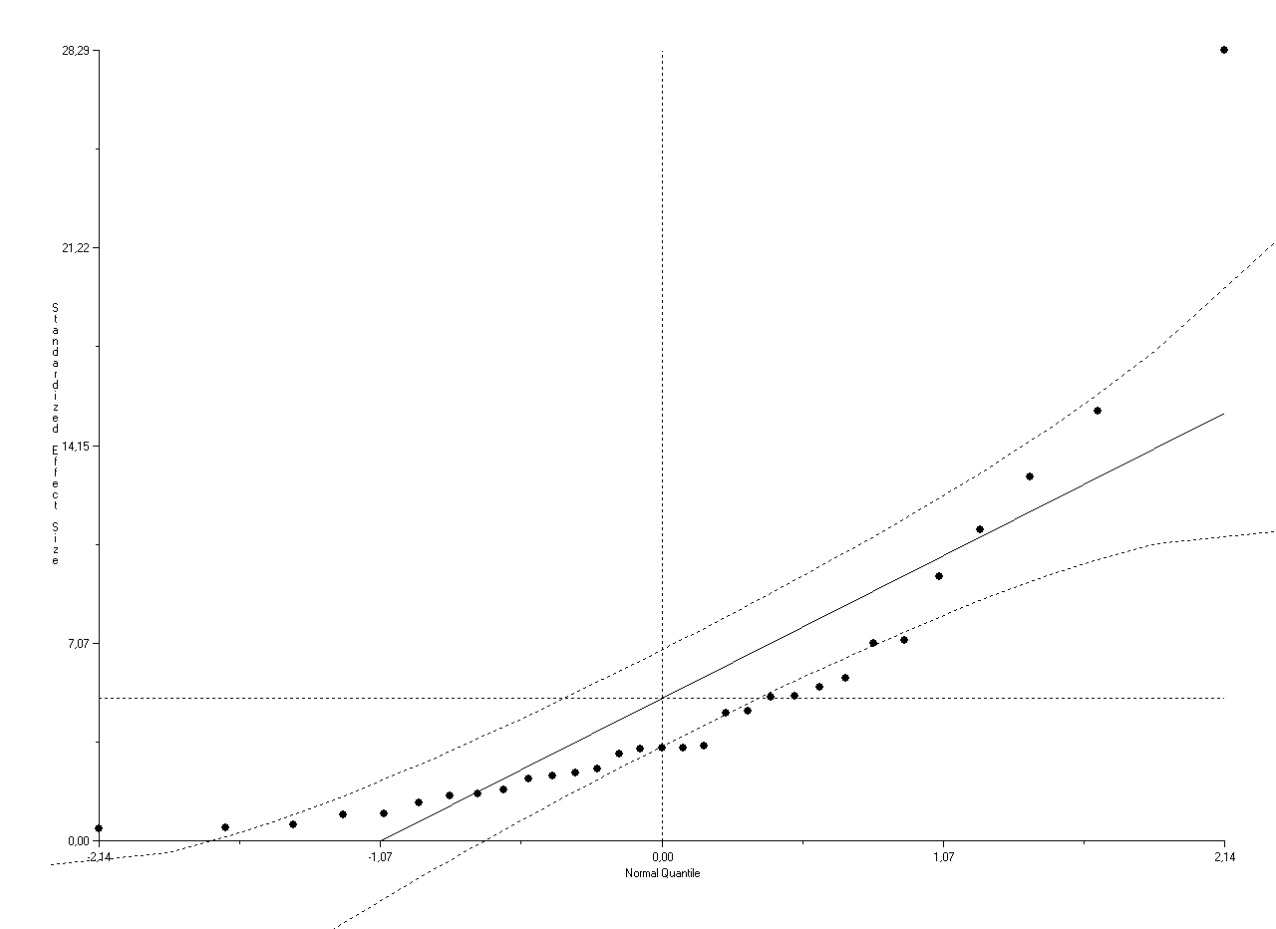
**Normal quantile plot for the ES(*z*) between impairment and health-related quality of life**. The plot compares two distributions by plotting there quantiles against each other. One of the distributions is the standard normal distribution (X as) while the other is the distribution one wishes to compare it against (here the Y as is defined as ES(*z*)_i_/√_i_). The deviations from linearity indicate deviations in normality. Publication bias will tend to leave strange gaps in the plot or lead to strange curves.

Review of the normal quantile plots (Figure 2 and 3) showed that one study [46] substantially deviated from normality. Removal of this study revealed statistical homogeneity of the datasets for the association between impairment and disability (Q statistics, *p *= 0.34), and between impairment and HRQL (Q statistics, *p *= 0.15).

**Figure 4:**
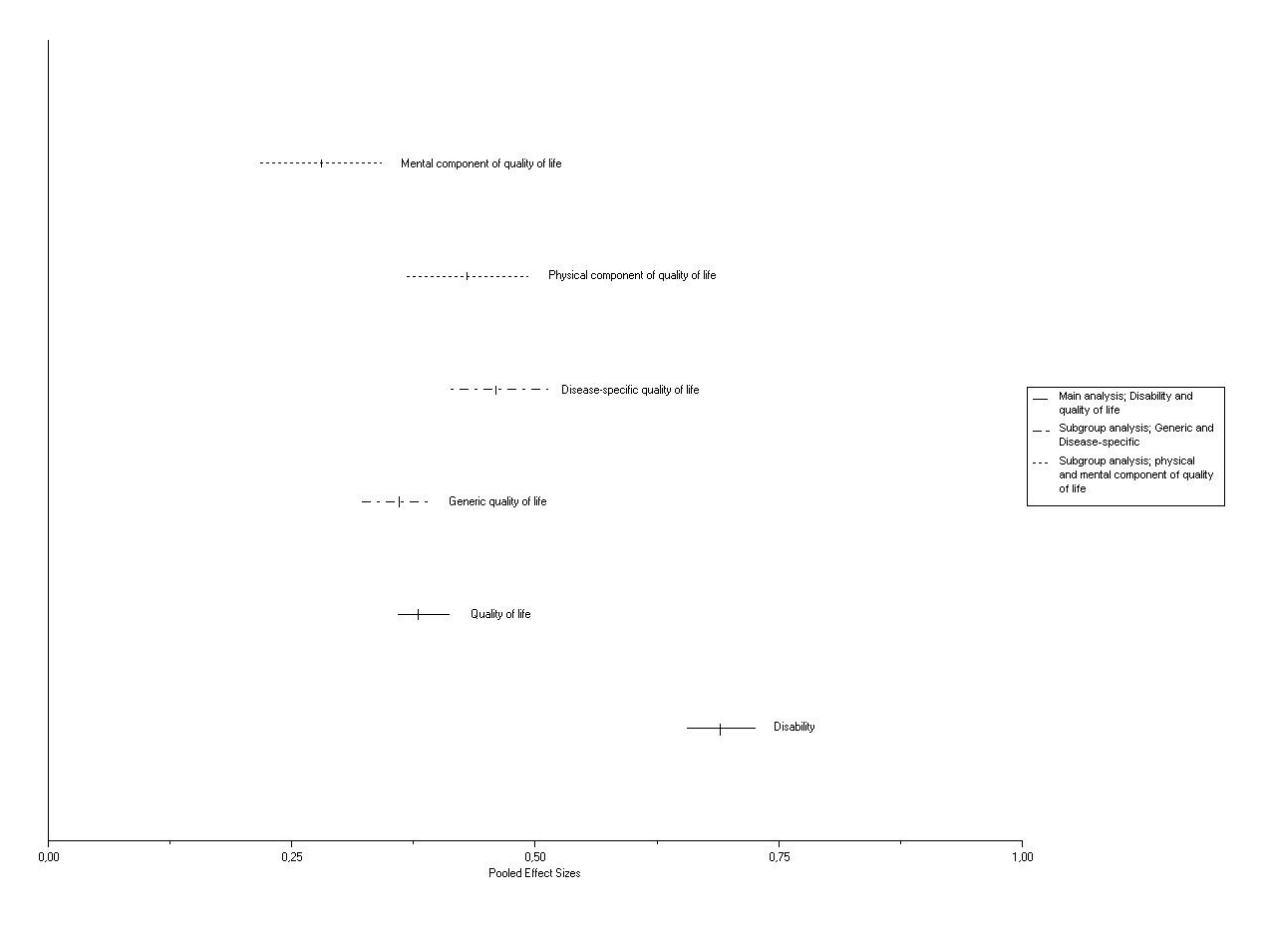
Meta-analysis for the ES(*z*) between impairment, disability, and health-related quality of life and the subgroup analyses.

On aggregate level (Figure 4), there was a moderate to large association between impairment and disability (pooled ES(*z*) = 0.69; 95% CI, 0.66 – 0.72). The aggregated relationship between impairment and HRQL was statistically significant smaller (pooled ES(*z*) = 0.38; 95% CI, 0.36 – 0.41). Backward transformation in correlation coefficients resulted in a moderate association between impairment and disability (r = 0.60; r^2 ^= 36%) and a weak association between impairment and HRQL (r = 0.36; r^2 ^= 13%).

### Subgroup analyses

Table 2 presents the ES(*z*) per study used for the subgroup analyses. Figure 4 also depicts the various impairment – HRQL subgroup analyses. No statistical heterogeneity could be demonstrated (range p-values of the Q statistics 0.35 – 0.44), with the exception of the association between impairment and the mental component of HRQL (*p *= 0.06).

The pooled ES(*z*) between impairment and generic HRQL measures was 0.36 (95% CI, 0.33 – 0.39; r = 0.35; r^2 ^= 12%) [16-22,24-27,29-32,34,37-42,45]. The pooled ES(*z*) between impairment and disease-specific HRQL measures was significantly higher (pooled ES(*z*) = 0.46; 95% CI, 0.40 – 0.51; r = 0.43; r^2 ^= 18%) [23,28,29,33,35,36,40,43,44,46]. Impairment measures were significantly higher related with the physical component of the HRQL measures (pooled ES(*z*) = 0.43; 95% CI, 0.39 – 0.47; r = 0.41; r^2 ^= 17%) [16,17,19,26,27,31,32,34,36,38-40,45] than with the mental component (pooled ES(*z*) = 0.28; 95% CI, 0.20 – 0.36 [random effects model]; r = 0.27; r^2 ^= 7%) [16,17,19-22,26-29,31,32,34,36-40,42,43,45].

### Publication bias

Review of normal quantile plots showed no clear indications for publication bias in both the analyses for the relationship impairment and disability and for impairment and HRQL (Figure 2 and 3). In addition, publication bias was not evident when the Spearman's rho rank correlation method was used (both *p *> 0.50). The fail-safe calculation by Rosenthal's method retrieved for both analyses a large number of studies (n = 27077/n = 9755) needed to be added in order to change the results of the meta-analysis.

## Discussion

In this study we investigated the impact of impairments on patients' functional health in a spectrum of diseases. The results of the meta-analysis show that impairments explain 36% of the variance of the disability scores and 13% of the variance of HRQL scores. Impairment scales account for 17% of the physical dimension of HRQL, but can explain no more than 7% of the psychological aspects of HRQL [47].

Due to the great variability in disease-related impairment scales and disability and HRQL instruments used in patient groups with a wide range of disorders, we expected heterogeneous data sets. Interestingly, after removal of one study which deviated from normality, homogeneity could be demonstrated in all analyses, except when we investigated the relationship between impairment and the mental component of HRQL. Possibly, this type of HRQL is less clearly defined compared to the other types of functional outcomes.

Treatments, such as new drugs or new surgical or radiological interventions, are aimed at reducing mortality and morbidity. If these treatments reduce impairments, the beneficial effects on functional health will be far more better detectable in the patient's level of disability than in his or her level of HRQL. Moreover, if the outcome is assessed in terms of HRQL, treatment effects will be more visible in the physical component than in the mental components, and will be better reflected in the disease-specific measures than in the generic HRQL measures. Therefore, outcome measures at the level of functioning of patients should be selected carefully when clinical studies are designed. It clearly does matter on which level the functional outcome measurement is chosen.

No doubt, HRQL scales represent an important measure from a patient's point of view, but have serious disadvantages in many clinical efficacy studies. The extent to which patients fulfill social roles and participate in their environment cannot be observed directly and depends not only on their functional ability, but also on personal traits, social circumstances and societal barriers. In particular, HRQL scores, which reflect the patient's perception of the consequences of disease, depend on numerous additional, usually psychosocial, factors other than the disease itself. This explains why the correlation between impairments and HRQL scores is weak, especially when emphasis is laid on subjective psychological scores of these scales. The subjective HRQL scores therefore are often beyond the influence of the physician. In the primary assessment of new drugs or new surgical procedures, there are many outcomes of interest and precisely defining them and measuring them will improve assessing the impact of new interventions. In contrast, disability (mobility, self-care) seem to be a more appropriate primary end point for assessing the functional health status of patients. The ability to perform basic and complex activities of daily life is, as we showed, not only better related to the disease process itself, but is also observable and predictive of dependence [48], and forms an essential aspect of the patient's HRQL [49], an aspect which is amenable for treatment.

A shortcoming of this meta-analysis might be that there are probably more studies than the 31 we found. Our search strategy was based on the ICIDH and ICF terms and few researchers distinguish between the terms impairment, disability and HRQL. If they distinguished between these terms correlations between impairment and disability or between impairment and HRQL were given rarely for all three domains together. Another point of discussion may be the concern for publication bias. However, the normal quantile plots, Spearman's rank correlation coefficient method and fail-safe calculation method do not give an indication for this type of bias. Finally and unfortunately, we were not able to perform subgroup analysis based on the methodological quality of the studies. This because the psychometric and clinimetric articles analyzed used a hotchpotch of research designs which made it impossible to formulate unequivocal methodological criteria in terms of, for example, appropriateness of the design chosen, sample size consideration, blind assessment of outcome parameters, statistical adjustment for confounders, or loss to follow-up [50].

## Conclusion

New drugs or new surgical or radiological treatments are developed from a pathophysiologic perspective and are primarily directed at reducing impairments. Traditionally, biological and impairment measures are preferred. Increasingly, it becomes evident that this type of outcome is not always relevant for patients [21,51]. Our study shows that patient's impairments are clearly better reflected in disability measures, than in HRQL instruments. The relatively low association between impairment and HRQL is found in patients with various disorders, therefore we conclude that to assess the efficacy of a new treatment, disability is an important functional endpoint in clinical studies.

## Competing interests

The author(s) declare that they have no competing interests.

## Authors' contributions

NW, RdH and MV initiated the project. NW and RdH screened, extracted and analysed the data. All authors participated in the discussion of the results, in the writing of the paper, and read and approved the final manuscript.

## Pre-publication history

The pre-publication history for this paper can be accessed here:


